# Development and cognitive testing of a food-frequency questionnaire to assess intake of plant-based protein foods among older adults

**DOI:** 10.1017/S1368980024002052

**Published:** 2024-10-21

**Authors:** Virginie Drolet-Labelle, Alexandra Bédard, Simone Lemieux, Vicky Drapeau, Lana Vanderlee, Danielle Laurin, Sophie Desroches

**Affiliations:** 1 Centre Nutrition, santé et société (NUTRISS), Université Laval, Québec, QC, Canada; 2 Institut sur la nutrition et les aliments fonctionnels (INAF), Université Laval, Québec, QC, Canada; 3 École de nutrition, Université Laval, Québec, QC, Canada; 4 Département d’éducation physique, Université Laval, Québec, QC, Canada; 5 Faculté de pharmacie, Université Laval, Québec, QC, Canada

**Keywords:** Older adults, Cognitive testing, Plant-based protein, FFQ, Think aloud approach

## Abstract

**Objectives:**

To develop a web-based food-frequency questionnaire (FFQ) measuring intake of plant-based protein foods (PBP) among older adults from the province of Quebec, Canada.

**Design:**

The questionnaire was adapted from an existing self-administered FFQ and first underwent expert panel evaluation for face and content validity. Then, three phases of cognitive testing were conducted in French, using the probe and think aloud approaches. Between each phase, the questionnaire was modified based on participants’ feedback to improve the clarity and comprehension of the questions.

**Setting:**

Quebec City, Quebec, Canada.

**Participants:**

Twenty adults aged 65 years and older participated. Purposive sampling was used to maximise variation in sociodemographic characteristics, including gender, age, education level and PBP consumption.

**Results:**

The expert panel found the twenty-eight-item questionnaire to be a comprehensive measure of PBP intake and suggested minor changes to improve its clarity. The cognitive interviews showed that our PBP-FFQ was generally well understood and identified issues requiring modifications to improve comprehension and accuracy.

**Discussion:**

Our FFQ provides a comprehensive measure of PBP intake, is well understood by older adults in Québec and will support rigorous assessment of PBP intake in this population but requires further validation to confirm its validity and reproducibility.

The health and environmental benefits of plant-based protein foods (PBP) are well documented. Indeed, PBP consumption has been associated with improved cardiovascular health^(1,2)^, lower all-cause mortality^(2,3)^ and a reduced environmental footprint compared to animal-based protein foods^(4)^. As a result, many food guides and dietary recommendations worldwide are now promoting PBP consumption^(5)^. For instance, the latest version of Canada’s food guide (CFG), published in 2019, recommends choosing PBP more often as an alternative to animal protein^(6)^ and the EAT-Lancet Commission’s proposed diet emphasises the consumption of plant-based foods^(7)^.

While adequate protein consumption is essential for healthy ageing, intakes tend to decrease with age and may fall below the recommendations^(8)^. For instance, according to the 2015 Canadian Community Health Survey, nearly 10 % of women aged 71 years and older in Canada had protein intakes below the Estimated Average Requirement, which is 0.66 g/kg per d^(8)^. Although several advantages of PBP over animal proteins have been raised by older adults, including better digestibility and affordability^(9)^, the vast majority of protein foods consumed by Canadian older adults in 2015 were from animal sources^(10)^. Greater inclusion of PBP in older adults’ diets could therefore contribute to this age group’s overall protein intake while adhering to current recommendations and promoting health. We recently conducted a study in the province of Quebec (Canada) in which we identified older adults’ barriers to introducing PBP, such as lack of knowledge about how to prepare these foods^(9)^, which will help inform future interventions to increase PBP consumption in the older adult population. To accurately assess the impact of such interventions, a questionnaire specifically designed for older adults that rigorously and accurately assesses PBP consumption is needed. To our knowledge, no such tool exists yet.

Dietary assessment in the older adult population presents several difficulties. Challenges with short-term memory tasks, for instance, can make 24-h recalls less suitable for this age group^(11)^. In addition, 24-h recalls often require significant technical or human resources and multiple administrations to better reflect habitual diet^(12)^. Longer questionnaires, such as diet histories or food recalls, may also be challenging for older adults, as they require more time and effort^(12)^. Therefore, shorter questionnaires may be more appropriate for older adults as they reduce the fatigue and burden associated with completion^(12,13)^. Although food-frequency questionnaires (FFQ) also rely on memory, several studies have shown that they are appropriate tools for assessing older adults’ food and nutrient consumption and for ranking older adults according to their intake^(14,15)^. Therefore, using a brief FFQ designed to specifically assess PBP intake may be appropriate for this population, especially since our focus is not on the total diet^(16)^.

Cognitive testing is a method for improving and adapting questionnaires by allowing respondents to verbalise their cognitive processes. For self-administered questionnaires, a pretesting step using probes and think aloud approaches can help identify problematic questions and understand how instructions and questions are interpreted^(17,18)^. Pretesting and refining a questionnaire based on feedback can also help improve the questionnaire to reduce the cognitive load associated with its completion^(19)^. Previous studies have demonstrated the effectiveness of cognitive testing in improving the clarity and comprehension of FFQ or other types of questionnaires^(20–22)^. The current study aimed to develop a web-based FFQ that specifically assesses the level of consumption of PBP in the older adult population from the province of Quebec, Canada. This paper describes the development process, the face and content validity evaluation by a panel of experts, and the cognitive testing conducted to assess whether the questions were clear and well understood by the target population.

## Methods

### Development of the questionnaire

An existing 136-item self-administered FFQ validated among adults from the province of Quebec, Canada^(23)^, was used to develop our web-based PBP-FFQ. The questionnaire was adapted by a graduate student in nutrition (VDL), and two researchers with expertise in questionnaire development (AB and SD). From this 136-item FFQ, the development team identified items related to PBP products and modified or added items to ensure that the questionnaire adequately represented the PBP products currently available in grocery stores in the Quebec market, particularly processed products, which were less captured in the original questionnaire. To properly estimate the amount of PBP consumed, foods with similar protein content were grouped in the same questions. This resulted in a twenty-six-item PBP-FFQ.

**Figure 1.**
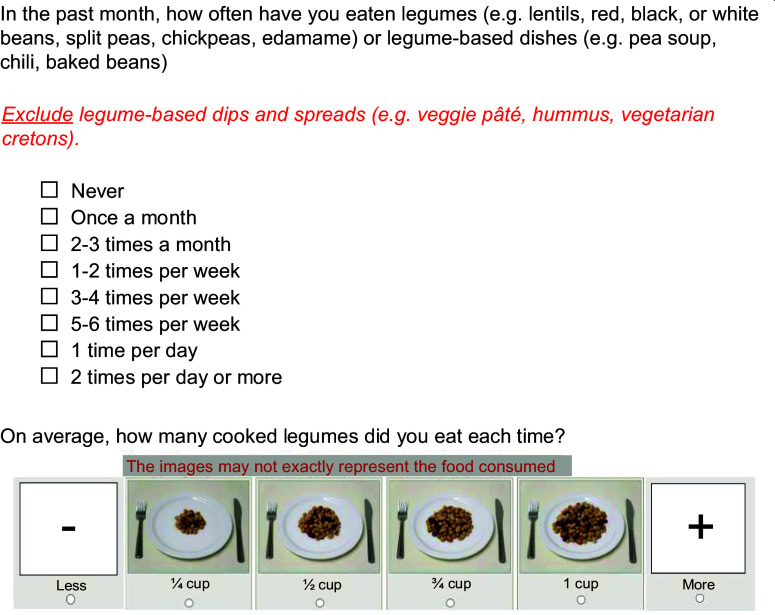
Question example (translated from the original French questionnaire).

The frequency of consumption of individual food items or groups of foods, in terms of day, week or month, remained the same as in the initial questionnaire (i.e. frequency of consumption in the past 30 d: never, once a month, 2–3 times per month, 1–2 times per week, 3–4 times per week, 5–6 times per week, 1 time per d and 2 times per d or more). Existing images from the 136-item FFQ were used to illustrate four different portion sizes, embedded within images of negative and positive signs to denote smaller and larger portion sizes, respectively. Figure 1 shows the visual of one of the questions. The images represented the exact food/food group questioned or a similar food item/food group, depending on the images available. All products were unbranded.

### Evaluation by an expert panel

An expert panel consisting of four researchers (SL, VD, LV and DL) with diverse conceptual and methodological expertise (PBP, questionnaire development and validation, cognitive testing and epidemiology), and with knowledge of our target population (older adults), evaluated the face and content validity of our initial questionnaire, to assess the extent to which the questions provided a good measure of PBP consumption, and whether the measures were well constructed and easy to understand. The development team presented the PBP-FFQ to the expert panel to obtain their general thoughts and comments. The revised questionnaire was then submitted to the experts for a more in-depth evaluation. Specifically, each expert was asked to evaluate whether the questions adequately and comprehensively measured PBP consumption, as well as the relevance and clarity of each question on a scale from 1 to 4. A score of 1 meant that the item was not relevant or not clear; 2, that it needed a major revision; 3, that it needed a minor revision and 4, that it was perfectly relevant or clear. To determine the content validity index, we calculated for each question the percentage of experts who rated the item’s relevance as 3 or 4, as suggested by Di Iorio (2005)^(24)^. A content validity index below 90 % suggests that the question should be modified or deleted^(24)^. The experts were also asked to provide their general comments on the instructions, answer options, question order and whether some questions should be added or removed.

### Cognitive testing interviews

#### Participants

Recruitment of participants began in June 2022. Newsletters featuring the project recruitment advertisements were sent to several older adults and retirees organisations and associations in the province of Quebec, Canada, as well as to participants from a study conducted by our team who agreed to be recontacted for another study^(9)^. Inclusion criteria were to (1) be 65 years of age or over, (2) reside in the province of Quebec, (3) live at home (i.e. not in a retirement home or long-term care) and (4) be able to read and understand French. Purposive sampling was used to maximise variation in sociodemographic characteristics, including gender, age, education level and PBP consumption, which ensured that a greater diversity of cognitive processes would be captured^(17)^. Specifically, we sought to recruit a sample that included as many men as women, with a well-balanced number in each age group (65–69 years, 70–74 years, 75–79 years and 80 years and older), and with at least one-third of the sample having not completed post-secondary education, as a proxy for lower literacy levels^(25)^. In addition, we sought to recruit a greater proportion of PBP regular consumers (PBP consumption in main meals ≥ 2 times /week) than PBP non-regular consumers (PBP consumption in main meals < 2 times/week), to obtain greater feedback on the consumption frequency options, the suggested serving sizes and the images used to represent food items/food groups. Participants did not receive compensation but were eligible to win one of the two $25 gift cards. The project was approved by the ethics committee of Université Laval (#2022-153/23-05-2022).

#### Procedure

**Figure 2.**
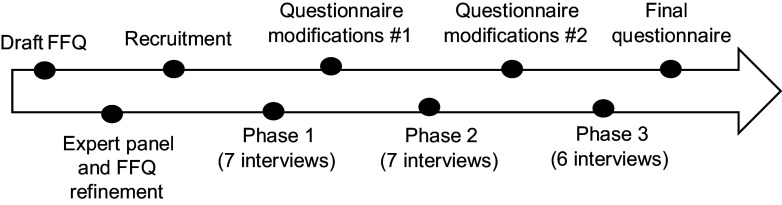
Development process.

Cognitive testing interviews were conducted in three phases from July to October 2022, which allowed for the refinement of our PBP-FFQ (see Fig. 2). Each phase of cognitive testing involved 6–7 participants. The interviews were conducted in French, and an observer was present at each interview to take notes and to discuss the participants’ behaviour and responses with the interviewer afterwards. Participants had the choice between an online Zoom and face-to-face interview at the research centre. Before starting the interview, participants gave verbal consent to the project and the video recording. Interviews were not transcribed, but recordings were available if needed.

After consent was obtained, we shared a screen with a PowerPoint presentation explaining the main purpose of the project, the concept of cognitive testing and the think-aloud approach^(18)^ using an example. The PBP-FFQ instructions were then given to the participant, followed by the PBP-FFQ questions. Each slide of the presentation covered one food item/food group, and the visual was representative of the future web-based self-administered questionnaire. For each food item/food group, participants were first asked about the frequency of consumption in the past 30 d and then about the serving size usually consumed. If participants selected ‘never’, they were not subsequently asked to report a serving size. Participants responded aloud and were encouraged to verbalise their thoughts by the interviewer who asked probes^(18)^ to elicit more information, such as: ‘How did you reach this answer? Are you confident of your answer? Did you have any difficulty answering this question?’. Finally, at the end of the interview, participants were asked if they consumed PBP that were not included in the PBP-FFQ to confirm its exhaustiveness, as well as general questions about the ease of completing the questionnaire, difficulties encountered, redundancy of questions and household situation.

### Analyses

After each interview, the interviewer and the observer met to discuss the participant’s challenges and identify potential changes that could be made to the questionnaire to address the issues identified. At the end of each phase, elements of the questionnaire and instructions were modified based on participants’ feedback and discussions with the development team. The modified elements were then carefully monitored in subsequent phases to assess improvements in understanding. When clarification was needed, recordings were reviewed to ensure an accurate understanding of the difficulties encountered.

## Results

### Experts panel

All questions were deemed relevant, with a score of 3 or 4 given by all experts. The content validity index score for each question was therefore 100 %, which is higher than the recommended 90 %^(24)^. All the experts considered the questionnaire to be exhaustive and did not suggest adding or removing any questions. However, the experts suggested some minor changes to the order, presentation and wording of the questions, to make the questionnaire easier to understand. Instructions were also slightly reformulated. Finally, to confirm if the reported intakes were representative of usual intakes, the experts recommended adding a question at the end of the PBP-FFQ to document whether a situation or event has significantly affected the participant’s eating habits in the past month: ‘In the past 30 d, did you have to make any changes to your usual diet for any reason (e.g. illness, travel, etc.)? (yes/no; if yes please specify)’.

### Cognitive testing

The three phases of cognitive testing conducted, for which we recruited a total of twenty participants (seven participants for phase 1; seven participants for phase 2; and six participants for phase 3), allowed for continuous improvement of the questionnaire. Only one of the twenty interviews was conducted in person and the others were conducted online. Participants’ characteristics are presented in Table 1. After the third phase of interviews, only few minor modifications were suggested by participants. Consequently, a fourth phase was not required. The issues raised during the three phases of cognitive testing were grouped into three themes, which are described below. Table 2 presents in more detail the changes made to the questionnaire.

**Table 1.** Sociodemographic characteristics

		*n*	%
Gender	Women	10	50
	Men	10	50
Age	65–69 years	7	35
	70–74 years	8	40
	75–79 years	3	15
	80 years+	2	10
Education	High school or less	7	35
	CEGEP^*^	3	15
	University	10	50
PBP consumption^†^	PBP non-regular consumers	5	25
	PBP regular consumers	15	75
Responsibility for meal planning and preparation	Always	16	80
	Often	4	20
	Sometimes	0	0
	Rarely	0	0
	Never	0	0
Household situation	Lives alone	12	60
	Lives with a partner	8	40

PBP, plant-based protein foods.

* CEGEP (*Collège d’enseignement général et professionnel)* offers pre-university and technical programmes and is unique to the province of Quebec.

† PBP non-regular consumers were defined as participants who consumed PBP less than twice a week at main meals, and PBP regular consumers as participants who consumed PBP two times or more per week at main meals.

**Table 2.** Summary of the issues and modifications

Category			Issue	Modification
Lack of clarity in the instructions	#1	PBP overall definition	Confusion over the definition of PBP was observed: At the end of the interview, some participants mentioned fruits, vegetables and/or grains when asked about PBP consumed but missing from the questionnaire. In the main questionnaire, some participants included vegetables (in French*: légumes*) in the legumes (in French*: légumineuses*) section. Questions about processed foods that could have a non-plant-based version were also confusing. For instance, some participants included dairy yogurt in the question about plant-based yogurt.	An exhaustive list of foods included and excluded from the questionnaire was added at the beginning of the questionnaire, specifying, for example, that fruits, vegetables, dairy products and grains are excluded from our definition of PBP. The list’s clarity was confirmed during phase 3.
	#2	Frequency and serving sizes	Consumption frequency had to be answered with closed response choices, which was challenging for some participants. Some had difficulty converting, for example, their usual consumption estimated at four times per month, into one time per week, which represented one of the choices. Others wanted to be precise in their answers, leading to more laborious responses.	At the beginning of the questionnaire, two complete examples of questions and answers that included specific elements that were identified as unclear, that is, one where a tofu recipe was consumed within 4 d in the same week (which corresponds to the answer choice: 1–2 times per week if no other tofu recipe was eaten in the month) and the other where a soya beverage was consumed twice in the same day (which required averaging the portion size), were added. The relevance of these examples was confirmed in the third phase. In the third phase, participants were also asked about their preference for answer choices (closed or open-ended, e.g. having the choice to indicate the frequency by day, week or month). Most preferred closed response choices (such as what was already proposed). Participants mentioned that the open-ended answer choices required more precision and thought, making the answers more difficult.
			The portion size normally consumed had to reflect an average of all intakes, which was also a challenge as some participants were reporting the sum of all intakes.	
Wording and content of the question	#3	Defining foods to include	Some questions were ambiguous as to which food(s) should be included. For example, there was considerable confusion between nutritional yeast and other types of yeast (e.g. brewer’s, bread and cake yeast), which could lead to overestimation of nutritional yeast intake.	A definition was added after phase 1, but it had to be modified after phases 2 and 3 because there was still confusion. The final definition was: *Nutritional yeast is a yellowish flake used, among other things, to season dishes*. Other definitions were added or adjusted to clarify the questions. Most were tested in at least one of the phases.
	#4	Defining foods to exclude	Some questions were ambiguous about which foods should be excluded. For example, the original question about pumpkin, sunflower and hemp seeds did not specifically exclude chia, flax and sesame seeds, and some participants included them.	A sentence specifying the exclusion of chia, flax and sesame seeds was added. Other exclusions were added or refined based on comments, and most were tested in at least one of the phases. After phase 1, to be more visible, exclusion statements were put in red, and the word ‘exclusion’ was bolded.
	#5	Combining two questions	Initially, two questions addressed the consumption of plant-based yogurt (regular and Greek). However, during the first phase, we found that none of the participants had consumed either of these foods and most were unfamiliar with these products. The term ‘Greek’ was also confusing because it was associated with animal-based yogurt.	The two questions on yogurt were grouped to avoid unnecessary questions and a checkbox was added to indicate the type of yogurt consumed (regular *v*. Greek), when applicable. This change was made after phase 1 and thereafter tested in phases 2 and 3.
	#6	Splitting one question into two questions	Initially, only one question grouped plant-based dips (e.g. hummus) and pâtés (e.g. veggie pâté and cretons), but one participant mentioned that these two foods are not consumed in the same way, making it difficult to indicate the frequency and amount consumed.	The question was therefore split so that veggie pâté and vegetarian cretons would each have their own question. This change was made after the third phase following a comment from a participant and was not validated.
	#7	Adding new questions	Some missing foods were highlighted by participants. For example, there were initially questions on regular tofu and soft tofu, but one participant brought up dessert tofu, which cannot be included in the other questions because of its lower protein content. Cooking soya cream was also identified as missing.	Questions on dessert tofu and cooking soya cream were added after phase 1.
	#8	Changing the order	The order of some questions could influence participants’ responses. For example, the questionnaire initially began with a question on legumes, which included several items (i.e. examples of legumes and legume-based meals) compared to other questions like tofu. Thus, the FFQ started with a difficult question that could surprise and discourage the participants.	In phase 3, the questions about tofu were moved to the beginning of the questionnaire, that is, before the question about legumes. Thus, the questionnaire started with an easier set of questions. The order of other questions was changed after phases 2 and 3, which also helped with the exclusion and inclusion issues, but only one change has not been tested with participants.
	#9	Adding precision to processed foods	Processed products *v*. homemade recipes could be a confusing element for participants in terms of what to include and exclude from each question. For example, some were unsure if they should include homemade legume patties in the burger patty question.	For questions related to processed products that have homemade versions, the term ‘commercial’ (in French: *du commerce*) was added to the question. Other questions were modified accordingly, in phases 2 and 3; only one change has not been tested with participants.
	#10	Increasing readability	Some questions were quite long, mainly because they contained examples or word repetition (i.e. oat *drink*, almond *drink* and rice *drink*) and were reported by participants as unclear and more difficult to understand. Some had to reread the question to fully understand it.	These questions were modified to reduce the number of examples and unnecessary words while ensuring that participants always know which food is being referred to. These changes were made after phases 1 and 2.
Issues related to the serving size	#11	Increasing readability	In line with the original questionnaire, various serving measurements were provided depending on the question, including millilitres, cups, measuring spoons, grams, ounces or units (e.g. sausages). Also, to facilitate responses regarding tofu and tempeh, measurement units using block proportion were added. However, some participants mentioned that the text under each image was too long which led to confusion and difficulty in answering, and some reported using only cups, spoons and block proportions.	The choices were simplified after phase 1 by removing the millilitres and ounces. To ensure that this did not affect the understanding of the question, in phases 2 and 3, participants were asked if they were comfortable with having the servings only in cups and spoons, and all agreed with this change. For the tofu and tempeh questions, the proportions (e.g. ¼ block) and the grams were kept as they were both used.
	#12	Making the ‘more’ and ‘less’ options more apparent	The ‘more’ and ‘less’ options were quite small, and some participants mentioned not noticing them. Indeed, even if their usual intake was below the smallest serving option, they did not choose ‘less’, which may lead to an overestimation of their consumption.	The plus and minus signs were enlarged to make them more visible and mentioned in the examples at the beginning of the questionnaire (see example 2). The former change could not be tested since it was made after phase 3.
	#13	Relevance of the pictures	The images used were from an existing FFQ. The most representative ones were selected which meant that they did not always represent the exact food. Many participants reported that the images helped them identify the servings consumed, but others mentioned that the quality of some images was not great. For example, the background of the image and the plate were white, and sometimes the food was also white (e.g. hummus, yogurt and seitan), which lacked contrast.	The images that lacked contrast or were not representative of the foods included in the question were identified. They will be modified in the final version of our questionnaire before validation.

PBP, plant-based protein foods.

#### Lack of clarity in the instructions

Some issues were raised regarding the clarity of the instructions. First, there was some confusion about the overall definition of PBP, which could lead to misinterpretation throughout the questionnaire. To address this ambiguity, a list of foods included and excluded from the PBP definition was added at the beginning of the questionnaire (see example 1 in Table 2). Second, some participants found it difficult to precisely report consumption frequency and serving size, especially because of the close-ended, multiple-choice style response options. Many wanted to be precise in their answers, leading to more laborious response processes. Therefore, at the beginning of the questionnaire, we included examples of responses to FFQ-PBP questions that addressed these issues (see example 2 in Table 2). Nevertheless, we asked participants in phase 3 which response option style they preferred, and closed response choices were indeed preferred to open response options. Finally, given the confusion between the number of times a food was purchased and the number of times it was consumed, a sentence was added to the instructions after phase 3 stating that the questions were about frequency of consumption, not frequency of purchase. This sentence will be added to each question in the final web questionnaire.

#### Wording and content of the questions

The cognitive testing revealed issues related specifically to the questions. First, participants were unsure about the types of foods that needed to be reported in some questions. We therefore added definitions and exclusions to these questions, making it easier to identify which foods should be included in their answers (see examples 3 and 4 in Table 2). Second, some questions were added, combined or split following participants’ feedback about incorrect groupings or missing foods (see examples 5, 6 and 7 in Table 2). Third, the order of some questions was changed to make the questionnaire more coherent, consistent and easier to complete (see example 8 in Table 2). Fourth, for processed products that have homemade and commercial versions, the word ‘commercial’ (in French: *du commerce*) was added to indicate the origin of the product and to reduce confusion (see example 9 in Table 2). Finally, the wording of some questions was changed to shorten the questions and to make them more readable for participants (see example 10 in Table 2).

#### Issues related to the serving size

Several changes have been made to the serving size sub-questions. First, to improve the readability and simplify the visual, we removed the amounts in ounces and millilitres, leaving only cups, teaspoons/tablespoons and grams (see example 11 in Table 2) and we confirmed with participants in subsequent phases that they were comfortable with this change. Second, the ‘more’ and ‘less’ options, which were initially quite small, were enlarged to improve their visibility (see example 12 in Table 2). It should be noted that serving size options were considered, for the most part, adequate. Only two questions required the modification of the serving size options with the addition of an intermediate option. Finally, although they were considered useful, the quality and representativeness of the images of certain food items/food groups were considered sub-optimal (see example 13 in Table 2), highlighting the need to create new images for some items.

#### Other comments and observations

When participants were asked at the end of the interview if they had experienced any difficulty in remembering what they had eaten in the past month, the vast majority reported no difficulty. Nonetheless, their answers still need to be validated. However, difficulties were raised regarding unfamiliarity with certain foods, particularly processed foods, which led to confusion among participants as to whether they had consumed the food in question. The addition of definitions resulted in less confusion and helped participants confirm that they had not consumed the food in question, and thus the frequency choice ‘never’ was selected. In addition, when asked at the end of the interview whether they had consumed any PBP other than those listed in the questionnaire, most participants responded that the questionnaire was exhaustive, but some mentioned non-PBP, reinforcing the need for the definition of PBP in the instructions (see example 1 in Table 2). We also decided to keep the question about missing PBP in the final PBP-FFQ, to ensure that all PBP are captured, including recently available products on the market that were not covered in the survey.

### Final version of the questionnaire

The final version of the PBP-FFQ included twenty-eight questions, in addition to the questions about missing PBP and about situations or events that could have influenced the participant’s eating habits in the past month and was presented to and approved by the expert panel. The final questions are shown in Table 3.

**Table 3.** Food items/food groups included in the final PBP-FFQ

Food group/item	Original question in French
1. Extra-firm, firm or semi-firm tofu	Tofu extra ferme, ferme ou mi-ferme
2. Soft or silken tofu	Tofu mou ou soyeux
3. Commercial dessert tofu	Tofu dessert du commerce
4. Tofu spread	Tartinade de tofu
5. Legumes (e.g. lentils, red, black, or white beans, split peas, chickpeas, edamame) or legume-based dishes (e.g. pea soup, chili, baked beans)	Légumineuses (par exemple, lentilles, haricots rouges, noirs ou blancs, pois cassés, pois chiches, edamames) ou mets à base de légumineuses (par exemple, soupe aux poids, chili, fèves au lard)
6. Plant-based protein dip (e.g. hummus)	Trempette à base de protéines d’origine végétale (par exemple, hummus)
7. Veggie pâté or vegetarian cretons	Végé-pâté ou cretons végétariens
8. Tempeh	Tempeh
9. Textured vegetable protein (TVP)	Protéine végétale texturée (PVT)
10. Seitan	Seitan
11. Peanut butter, nut or seed butter (e.g. almond, sesame, cashew or sunflower seed butter)	Beurre d’arachides, beurre de noix ou de graines (par exemple, beurres d’amandes, de sésame, de noix de cajou ou de tournesol)
12. Pumpkin, sunflower or hemp seeds	Graines de citrouilles, de tournesol ou de chanvre
13. Chia, flax or sesame seeds	Graines de chia, de lin ou de sésame
14. Peanuts or nuts (e.g. almonds, peanuts, walnuts, cashews, pecans, pistachios)	Arachides ou noix (par exemple, amandes, arachides, Grenoble, cajous, pacanes, pistaches)
15. Meatless sausages	Saucisses sans viande
16. Sliced meatless deli meats that imitate, for example, ham, bologna or bacon.	Charcuteries tranchées sans viande qui imitent, par exemple, le jambon, le bologne ou le bacon.
17. Pre-formed commercial burger patties made from plant-based proteins (e.g. Beyond Meat or Yves brand)	Galettes à burger préformées du commerce à base de protéines d’origine végétale (par exemple, de marque Beyond Meat ou Yves)
18. ‘Meatless ground’ (e.g. Yves or Gardein brand)	« Sans-viande haché » (par exemple, de marque Yves ou Gardein)
19. Commercial meatless nuggets that imitate, for example, the taste of chicken or fish	Croquettes du commerce sans viande qui imitent, par exemple, le goût du poulet ou du poisson
20. Meatless chicken (e.g. Tofurky brand)	Végé-poulet (par exemple, de type Tofurky)
21. Soya beverages	Boissons de soya
22. Almond, oat or rice-based plant beverages	Boissons végétales à base d’amandes, d’avoine ou de riz
23. Cooking soya cream (e.g. Belsoy brand)	Crème de soya à cuisson (par exemple, de type crème Belsoy)
24. Dairy-free plant-based cheese	Fromage végétal sans produits laitiers (fauxmage)
25. Dairy-free plant-based yogurt (e.g. soya, oat, almond or coconut-based)	Yogourt végétal sans produits laitiers (par exemple, à base de soya, d’avoine, d’amandes ou de noix de coco)
26. Commercial dairy-free pudding (e.g. Belsoy soya pudding)	Pouding végétal du commerce sans produits laitiers (par exemple, pouding de soya Belsoy)
27. Nutritional yeast	Levure alimentaire
28. Spirulina	Spiruline
In conclusion, are there any PBP not mentioned in the questionnaire that you consumed in the last month?	Pour conclure, y a-t-il des aliments protéinés d’origine végétale non mentionnés dans le questionnaire que vous avez consommés au cours du dernier mois ?
In the past 30 d, did you have to make any changes to your usual diet for any reason (e.g. illness, travel, etc.)?	Au cours des 30 derniers jours, est-ce que pour une raison quelconque vous avez dû apporter des modifications à votre alimentation habituelle (par exemple lors d’une maladie, d’un voyage, etc.)?

PBP, plant-based protein foods.

## Discussion

This study is the first to detail the development process of a FFQ focused on PBP and tailored to the older adult population. The three phases of cognitive testing conducted with adults over 65 years old in the province of Quebec resulted in a final PBP-FFQ consisting of twenty-eight questions that may support a better assessment of the amount and frequency of PBP consumed by older adults. Feedback from our expert panel suggested that the questionnaire was comprehensive and relevant and allowed us to improve the clarity of the questions asked. Cognitive testing with our target population further enhanced the comprehensiveness of the questionnaire, confirmed its exhaustiveness and played a key role in improving the FFQ-PBP. Indeed, feedback from participants allowed us to make progressive changes that would otherwise have led to misreporting of food consumption, which could have jeopardised the accuracy of the future questionnaire. By reporting aloud the difficulties they encountered, participants highlighted which elements of the instructions and questions were ambiguous, allowing us to clarify them. Using probes also helped us to gain a deeper understanding of participants’ responses and to identify questions that had been misunderstood, unbeknownst to the participants. These findings highlight the value of cognitive testing in identifying small changes that can have a major impact on questionnaire comprehension and accuracy. Overall, the PBP-FFQ was well understood by older adults. Most changes were minor and were tested in at least one phase following their modification. The issues raised were mainly related to comprehension, for example, instructions, wording, content of the question and serving size sub-questions. Most participants also indicated that the questionnaire was not cognitively demanding in terms of their ability to recall information or the length of the questionnaire. However, whether the questionnaire can adequately report the correct amount and frequency of PBP consumed has yet to be validated.

Many of our modifications were similar to other studies that used cognitive testing. For example, Hutchison *et al.* also raised the issue of lack of clarity as to which foods to include and exclude in each question in their screener assessing adults’ alignment with the 2019 CFG recommendations. To address this issue, they added details and changed the sequence of some questions, which improved the clarity of their questionnaire^(20)^. In the current PBP-FFQ, the definitions and exclusions proved to be very effective and allowed participants to quickly know whether the item had been consumed or not, especially for ambiguous questions such as the one about nutritional yeast shown in Table 2. Similar to our study, Subar *et al.*, who conducted cognitive testing with older adults, found that open-ended response choices in FFQ were not ideal for self-administered questionnaires and could lead to errors, compared to closed-ended choices^(22)^. This suggests that closed-ended response choices are likely to be preferred in this population. Other authors have also shown that cognitive testing can be useful in identifying unfamiliar concepts/words for participants^(21)^. In our case, some PBP or foods derived from them were unknown to the participants, which justified the addition of definitions. However, our results contrast with a previous study in which older adults reported lower serving sizes than younger individuals, suggesting that portion size adjustments are needed in FFQ^(26)^. Indeed, although our PBP-FFQ was developed based on a questionnaire validated in the adult population, we did not have to adjust the serving sizes downwards.

This project has several strengths. Our approach used both an expert panel and cognitive testing with our target population, resulting in a more thorough improvement of our questionnaire. We used the think aloud and probing approaches, which allowed for an in-depth study of participants’ thinking processes. Also, the baseline FFQ had been previously validated in the adult population of Quebec, and our sample was diverse in terms of sociodemographic characteristics which ensured that we captured a diversity of cognitive processes and literacy levels.

Our project also has some limitations. First, some of the foods in our questionnaire, mostly processed foods (i.e. meatless chicken, meatless nuggets, etc.), were not consumed by any of the participants in the cognitive testing. As a result, we were unable to determine if the serving sizes were appropriate for these foods. Nevertheless, these serving sizes were inspired by those of comparable foods already included in the PBP-FFQ, which may suggest that they are adequate. Second, the foods included in this questionnaire were representative of what was on the market in 2022, but the availability of PBP products is constantly evolving, which may result in missing foods and consequently in an underestimation of PBP intakes. However, adding the question inviting participants to report foods not included in the questionnaire could help in this regard, allowing us to adjust our questionnaire over time. Third, it should be noted that completing this type of questionnaire requires memory skills^(12)^. As we conducted our cognitive trials among individuals with no apparent cognitive impairment, the generalisability of our results and use of this questionnaire with individuals with cognitive decline may be limited. A different type of dietary assessment may be necessary for this population. Our sample also consisted of volunteers who were responsible for meal planning, potentially introducing a bias as they might have a heightened interest in food and nutrition. Results may differ in other population subgroups. Additionally, the generalisability of our findings may be limited to individuals within the province of Quebec, as the questionnaire is in French and the availability of PBP in grocery stores may differ elsewhere. Fourth, we made some minor changes to the questionnaire after the final round of testing, which we were unable to evaluate (i.e. more representative images, improved serving sizes, splitting of one question and other minor changes in question wording and exclusions). However, these changes were suggestions from participants to clarify some minor remaining issues and did not represent significant changes to the questions.

## Implication for research and practice

This PBP-FFQ represents a potential tool for assessing PBP consumption among older adults and, ultimately, for evaluating the effectiveness of an intervention. Compared to a comprehensive FFQ assessing total dietary intake, its concise format makes it easier for older adults to complete. In addition, the cognitive testing we conducted improved the understanding of the tool among this population, further increasing the reliability of the data collected. This step was particularly relevant considering their lack of familiarity with PBP^(9)^ and the decline in protein consumption with age^(8)^. Our PBP-FFQ can also help researchers, policymakers and healthcare providers better understand the current state of PBP consumption among older adults and help develop targeted interventions to improve consumption. Finally, as we are still in the development phase, further validation steps, including comparison of our final PBP-FFQ to a food record and assessment of its reproducibility, will be necessary to complete the validation process.

## Conclusion

A comprehensive twenty-eight-item PBP-FFQ was developed through a rigorous process that included collaboration with an expert panel and cognitive testing with older adults. A validation study assessing the reliability and validity of our PBP-FFQ will be conducted as the next step. The development of such a questionnaire will support rigorous evaluation and monitoring of the PBP consumption habits among older adults.
